# The impact of organizational characteristics on the outcomes of septic patients admitted to the ICU

**DOI:** 10.1186/2197-425X-3-S1-A473

**Published:** 2015-10-01

**Authors:** L Sarmet Cunha Farah Rabello, F Bozza, J Kahn, P Brasil, D Angus, J Salluh, M Soares, ORCHESTRA Study Investigators

**Affiliations:** 1 Instituto Nacional de Cancer, Rio de Janeiro, Brazil; 2 D'Or Institute for Research and Education, Rio de Janeiro, Brazil; 3 University of Pittsburgh Medical Center, Pittsburgh, PA USA

## Introduction

Critically ill septic patients have high rates of death, complications and resource use, mainly in emerging countries.

## Objectives

To investigate the impact of organizational factors on the outcomes of septic patients in a large sample of Brazilian ICUs.

## Methods

Retrospective cohort study in 6,779 patients with severe sepsis (n = 5,294, 78%) or septic shock (n = 1,485, 22%) directly admitted from the emergency department to 69 ICUs (private hospitals, n= 62(90%); medical-surgical; n= 57(83%)) during 2013. We retrieved demographic, clinical and outcome data from an electronic ICU quality registry (Epimed Monitor System). We surveyed ICUs using a standardized questionnaire regarding organizational aspects, staffing patterns and process of care. We used multilevel logistic regression analysis to identify factors associated with hospital mortality.

## Results

38 (55%) ICUs had critical care training programs. Median graduate nurse-bed ratio was 0.23 (IQR, 0.18-0.29) and board-certified intensivists were present 24/7 in 16 (23%) of ICUs. Routine clinical rounds occurred in 59 (86%) and daily checklists were used in 34 (49%) ICUs. Sepsis protocol was implemented in 83% ICUs. The most frequent sources of infection were pulmonary (3,536 (52%)) and urinary tract (1,283 (19%)). Invasive mechanical ventilation was used in 1,373 (20%) patients and dialysis in 289 (4%) patients. SAPS 3 score was 54 (47-62) points. ICU and hospital mortality rates were 19% and 26%. Adjusting for relevant patients' characteristics (SAPS 3 score, admission diagnosis, chronic health status, comorbidities, MV use), case-volume and type of ICU, average graduate nurse/bed ratio during shifts (OR = 0.254 (95%CI, 0.068-0.945)) and the number of other implemented protocols (OR = 0.932 (0.862-1.008)) were associated with lower mortality. In a second model, the presence of intensivist nurses 24/7 in the ICU (OR = 0.600 (0.390-0.924)) was also associated with better outcomes.

## Conclusions

Nurse staff patterns and the implementation of clinical protocols were associated with mortality in septic patients and are potential targets to improve care of these patients in emerging countries.

